# CT perfusion in acute stroke

**DOI:** 10.4103/0971-3026.43837

**Published:** 2008-11

**Authors:** Niranjan Khandelwal

**Affiliations:** Department of Radiodiagnosis and Imaging, PGIMER, Sector 12, Chandigarh, India

**Keywords:** Stroke, CT perfusion, cerebral, ischemia

## Abstract

Stroke is a heterogeneous syndrome caused by multiple mechanisms, all of which result in disruption of normal cerebral blood flow and thereby cause cerebral dysfunction. Its early diagnosis is important as its treatment is dependent on the time elapsed since ictus. Delay in diagnosis and treatment translates into increase neuronal loss and thereby increased morbidity. CT scan, and in particular perfusion CT, has helped greatly in the early diagnosis of stroke. This article is an endeavor to explain the pathophysiology of cerebral ischemia and the role of CT perfusion in detecting it.

## Introduction

Stroke is a heterogenous syndrome caused by multiple disease mechanisms, all of which result in disruption of normal cerebral blood flow, causing derangements of cerebral function.

Broadly, stroke can be classified into two categories: ischemic and hemorrhagic. The former accounts for an estimated 80–85% of cases; the rest are hemorrhagic.[1] Stroke is a leading cause of death in developed countries and one of the most common causes of long-standing disability. Atherosclerosis of the carotid arteries is by far the most common predisposing condition for stroke.

It is important to understand the normal physiology of the brain for accurate interpretation of stroke imaging. The brain is continuously perfused with blood during systole as well as diastole, with 15–20% of the total cardiac output going to the brain. Cerebral blood flow is approximately 800 ml/min. This high and continuous blood flow is necessary as the brain uses glucose, exclusively, for energy metabolism and is unable to store energy. Cerebral blood flow is equipped with an autoregulatory mechanism, which protects against hypoxia and low perfusion. It is a multifactorial mechanism, involving neurogenic, myogenic, and metabolic controls. This autoregulation tries to maintain a mean arterial pressure of 60–100 mm Hg and a cerebral blood flow of 50–60 ml/100 gm of brain per minute. When the cerebral blood flow decreases, the autoregulatory mechanism tries to compensate by increasing the blood pressure and inducing vasodilatation. However, if the blood flow decreases so much that it falls below a critical level, infarction results. Table 1 gives the relative values of cerebral blood flow and their effect on cerebral tissue.[2,3]

**Table 1:** Cerebral perfusion and corresponding blood flow levels

Brain perfusion	Cerebral blood flow
Normal	>50–60 ml/100 gm/min
Oligemic	30–60 ml/100 gm/min
Ischemic	20–30 ml/100 gm/min
Infarction	< 10 ml/100 gm /min

## Aims of Imaging

Stroke imaging serves two purposes: first, to diagnose or confirm the occurrence of a stroke and, second, to assess the amount of potentially salvageable brain tissue and irreversibly infarcted tissue; both are necessary, the first for planning management strategy and the second for prognostication. CT perfusion (CTP) is a tool that has been successfully employed to assess the extent of salvageable tissue.

It is important to understand the meaning of potentially salvageable tissue. Whenever there is a decrease in the flow of blood to a particular area of the brain, collateral supply from the leptomeningeal vessels and from normal surrounding vessels tries to compensate. This results in a central area, the infarct, which receives little or no blood supply and a larger peripheral area where autoregulatory compensation tries to ensure the maintenance of adequate blood supply. This peripheral area is potentially salvageable by thrombolytic therapy and is called the ‘penumbra.’[4,5]

According to Rowley,[6] the imaging of stroke involves the evaluation of four ‘P’s, namely parenchyma, pipes (cerebral vessels), perfusion, and penumbra.

### i) Evaluation of parenchyma:

This is done by acquiring a nonenhanced CT scan of the brain. We usually acquire 5-mm sections of the posterior fossa and 10-mm sections beyond that. The primary role is not to diagnose ischemic stroke or infarction but to identify early signs of ischemia and, more importantly, to rule out out hemorrhage, as this has important treatment implications. Also, conditions that can clinically present with the stroke syndrome, e.g., acute meningitis, subarachnoid hemorrhage, and neoplasm, can be ruled out.

**Figure 1 (A–E):**
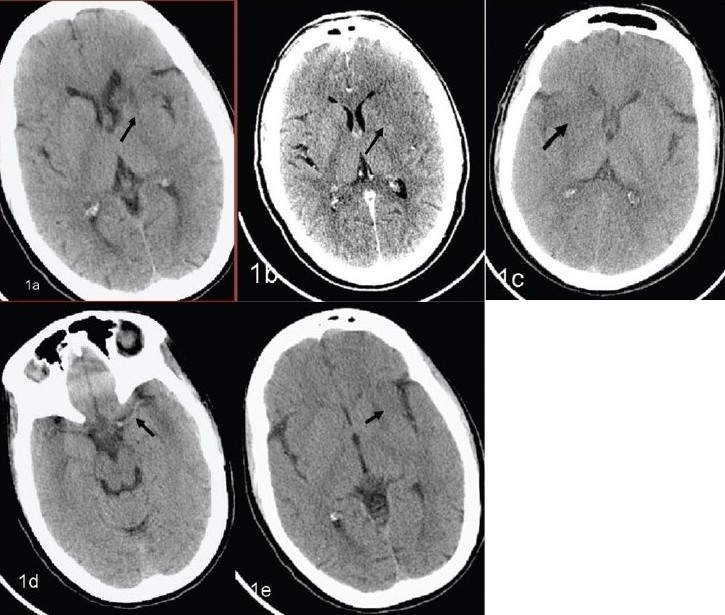
Early signs of infarction. Multiple NCCT scans show obscure lentiform nucleus (arrow in A), loss of gray–white matter interface (arrow in B), hypodensity in the lenticular nucleus (arrow in C), a dense middle cerebral/basilar artery sign (arrow in D), and loss of the insular ribbon (arrow in E)

Noncontrast computed tomography (NCCT), if carefully evaluated, can reveal some subtle signs of early stroke; for example:

1. Obscure lentiform nucleus [Figure 1a]
2. Loss of gray–white matter interface [Figure 1b]
3. Hypodensity in the lenticular nucleus [Figure 1c]
4. Dense middle cerebral/basilar artery sign [Figure 1d]
5. Loss of insular ribbon [Figure 1e]

The limitation of an NCCT scan is that up to 40% of stroke patients have a normal scan in the first few hours and even if some of the subtle signs enumerated above are present, the observer may still miss them.[7] MRI imaging, especially diffusion-weighted imaging (DWI), scores over CT scan in this respect as DWI is very sensitive in detecting early ischemia and can detect changes as early as 20 min after the onset of stroke.[8,9]

### (ii) Evaluation of pipes (cerebral blood vessels):

CT angiography (CTA) is an advanced application of present-day multislice spiral CT scan machines. It allows the comprehensive evaluation of arteries anywhere in the body.

The protocol followed in our department for angiography of the neck and cerebral vessels is as follows [Table 2]: 100 ml of iso-osmolar nonionic contrast is injected at the rate of 5 ml/s.

**Table 2:** CT angiography protocol

Parameters	Neck	Brain
Range	Arch of aorta to base of skull (BOS)	BOS to vertex
Scan	Helical	Helical
Slice thickness	2.5 mm	1.25nmm
Interval	1.25 mm	0.63 mm
Speed	7.5	3.75
Pitch	1.5:1	0.75:1
kV	120	120
mA	280–300	280–300
Rotation time	0.6 s	0.6 s
FOV	35 cm	25 cm

**Figure 2:**
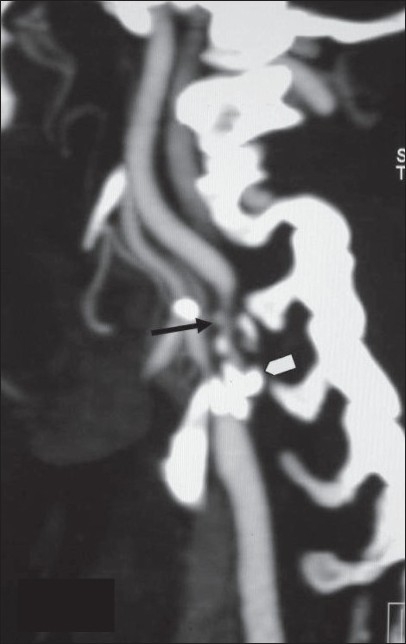
Saggital MIP image of a CT angiogram shows significant ICA narrowing (arrow) due to a calcified plaque (arrowhead) in the bulb and at the origin

**Figure 3:**
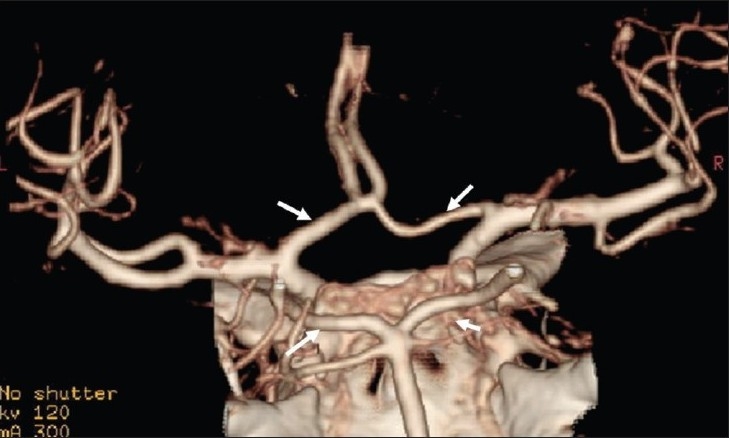
Intracerebral vessels (circle of Willis) seen in detail on VR image

This is useful in the assessment of stenosis or occlusion of the carotid arteries [Figure 2] or vertebral arteries in the neck, which can act as predisposing factors for a stroke. Also, the evaluation of intracranial arteries [Figure 3] is possible with a high degree of accuracy. CTA is now gradually replacing digital subtraction angiography (DSA) for this purpose. It is however important to stress the need for evaluation of the source or base images along with the reconstructed images as any kind of reconstruction is operator dependent and there is some inherent loss of information in these techniques.

### (iii) Evaluation of perfusion and penumbra:

Both these terms are interrelated. ‘Penumbra’ is the region surrounding the infarcted core where the damage is still reversible. It is the aim of functional imaging, whether it be CTP, MR perfusion, or SPECT imaging, to differentiate between the infarcted core and the ischemic penumbra so that measures can be taken to reverse the ischemic changes and salvage as much brain tissue as possible.

## CTP Evaluation

CTP provides absolute and relative information about brain perfusion parameters, namely cerebral blood flow (CBF), cerebral blood volume (CBV), mean transit time (MTT), and time to peak (TTP). MTT is the time between the arterial inflow and the venous outflow. TTP refers to the time taken by the contrast to achieve maximum enhancement (HU value) in the selected region of interest (ROI) before its value starts decreasing. CBV is the volume of blood available per unit of brain tissue and is usually measured as milliliters per 100 gm of blood.

### (a) Change in CTP parameters in ischemic brain:

With decrease in CBF due to any cause, cerebral autoregulation ensures adequate CBV by causing capillary dilatation which, in effect, causes increase in MTT and CBV. This continues till the decrease in CBF reaches a critical level (usually 20% of its normal value), at which point autoregulation fails and there is reduction in CBV and CBF. CTP, by measuring these values, tries to identify how much area of the brain is ischemic and/or infarcted. In general, if CTP shows a decrease in CBF with a stable or increased CBV, it signifies reversible ischemia; if both CBF and CBV fall below a critical level, it signifies irreversible infarction.[10,11]

### (b) Data acquisition:

**Figure 4:**
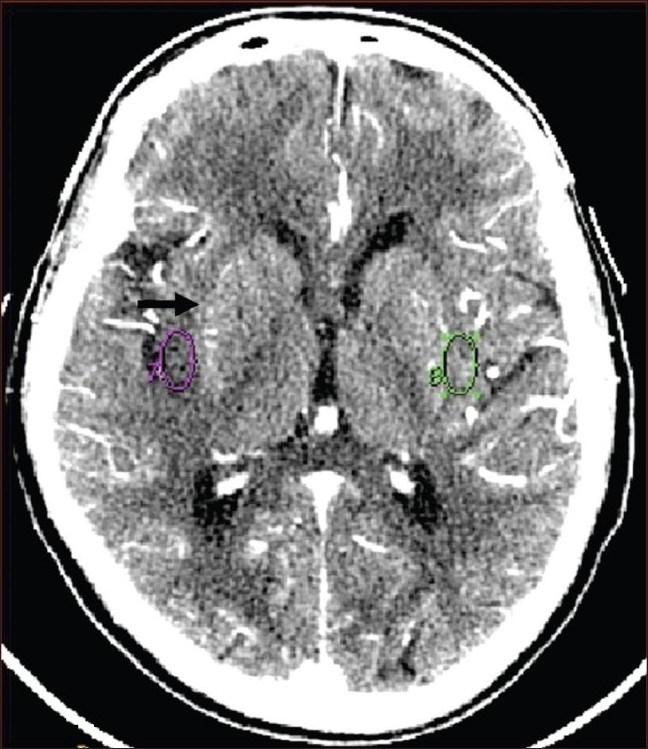
CTP data acquisition is done at the level of basal ganglia

Present-day multislice CTs do not allow for whole-brain perfusion assessment. Thus, if an anterior circulation infarct is suspected, data acquisition is done at the basal ganglia level [Figure 4] and if a posterior circulation infarct is likely, then at it is done at the level of the mid-cerebellum. Depending upon the type of scanner, one can take 1–4 sections of 5–10 mm thickness at the levels mentioned. The protocol used in our department is given in Table 3.

**Table 3:** CT perfusion protocol

Scan type	Cine mode
Slice thickness	5 mm
Interval	0
Range	4 Contiguous 5-mm Sections
kV	120
mA	200
Gantry angle	Parallel to orbital roof
DFOV	25 cm
Duration	45 s
Prescan delay	5 s
Contrast	50 ml at 5 ml/s

### (c) Postprocessing:

**Figure 5:**
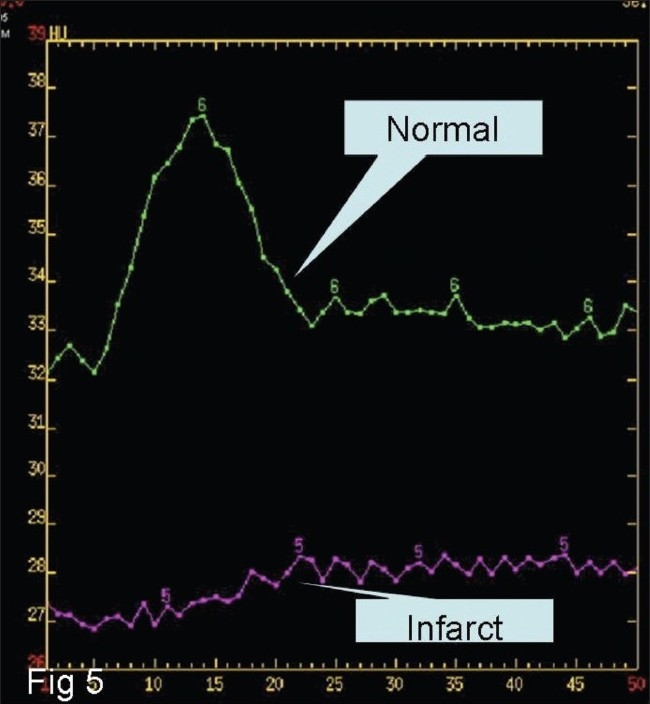
Time–attenuation curve of CBF showing normal flow (upper curve) and significant reduced flow in the infracted region (lower curve)

Postprocessing of the data is done by using specialized software. It involves confirmation of certain parameters which are automatically chosen by the software, and it generates color perfusion maps as well as time–attenuation curves (TAC) from which TTP/MTT, CBV, and CBF can be calculated for any area of interest [Figure 5].

### (d) Data interpretation:

This can be done qualitatively and quantitatively. For rapid assessment and a gross overview color-coded maps can be used to look for large ischemic areas; the method is reported to have a sensitivity of 90%.[12] However, quantitative assessment is done by placing multiple ROIs in the suspected ischemic area and at the corresponding location in the contralateral lobe. The relative values of percentage reduction, as well as absolute values in the regions under the ROI, can then be obtained. These values can then be used for stroke evaluation.

It is difficult to assign a specific threshold value for any of the CTP parameters below, which one can say with certainty that the ischemia is reversible or irreversible. No definite values can be assigned to this decrease in flow and volume. Wintermark *et al.*, however, have quantified it and observed that cerebral pixels with a decrease of 34% of blood flow or more as compared to the normal side are ischemic, and the ischemic pixels with blood volume below 2.5 ml/100 gm indicate infarcted tissue.[13]

A systemic approach for evaluation of these parameters is described below.

**Figure 6:**
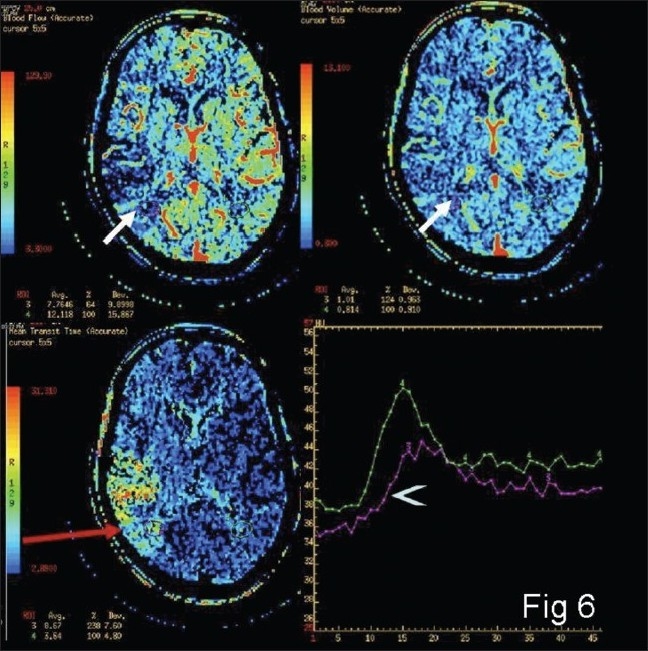
Color-coded maps from the penumbra (arrows) showing mild reduction in the flow as compared with the normal side (arrow head)

**Figure 7:**
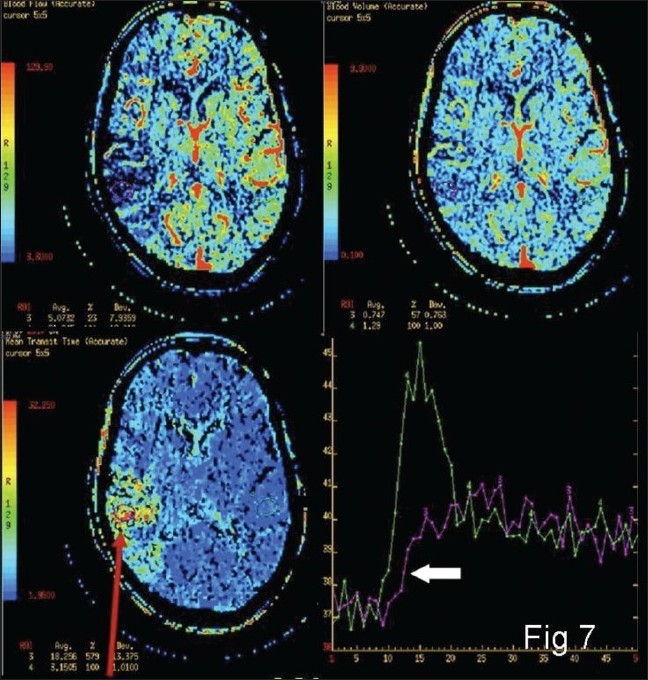
Color-coded maps and time–attenuation curve (bottom right panel) from the umbra (arrow) showing significant reduction in the flow as compared with the normal side (block arrow)

MTT, CBV, and CBF values from multiple areas under consideration on the affected as well as the normal hemisphere are obtained first. Then the MTT values are evaluated. If the MTT is raised as compared to the normal side, ischemia/infarction is present. Next, to differentiate ischemic and infarcted tissues, CBF and CBV values are compared. If CBF is reduced and CBV is normal or slightly reduced, the tissue ischemia is likely to be reversible [Figure 6]; if CBF and CBV are markedly reduced or if TTP is not measurable, the tissue may be infarcted [Figure 7].

**Figure 8 (A–F):**
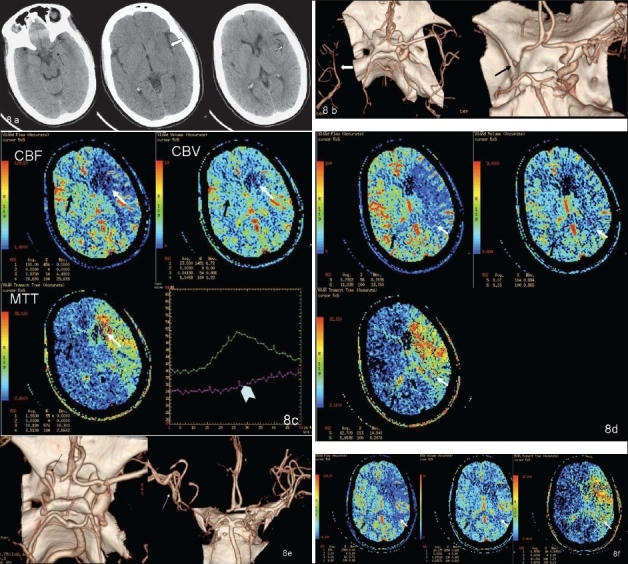
Initial NCCT (A) (at 1.5 h from stroke onset) showing hyperdense left MCA (black arrow), loss of insular ribbon (white open arrow), and obscuration with subtle hypodensity of the left lentiform nucleus (curved arrow). CTA volume-rendered images (B) show complete occlusion of the left intracranial ICA and M1 segment (black arrow on the right); note poor filling of the distal MCA branches, possibly through leptomeningeal collaterals (white arrow in the left image). Color-coded perfusion maps (C) for CBF, CBV, and MTT- ROI (white arrow) placed in the ischemic core—umbra—with mirror ROI (black arrow) in the contralateral hemisphere; relative values for CBF, CBV, and MTT are shown as a percentage of normal hemispheres (left hand bottom corner of each map). Time–attenuation curve showing lack of contrast arrival peak in the ROI on the affected side (arrow head). Color-coded perfusion maps (D) for CBF, CBV, and MTT- ROI (white arrows) placed in the ischemic penumbra, with mirror ROI (black arrows) in the contralateral hemisphere. The values (left hand bottom corner of each map) of rCBF and rCBV are greater, and rMTT lesser, than those in the umbra (see C). Post-thrombolysis CTA volume-rendered image (E) showing partial recanalization of the left cavernous ICA (black arrow) with occluded supraclinoid ICA and M1 segment and better filling of distal MCA branches (white arrow) as compared to the pre-thrombolysis CTA (see B). Color-coded perfusion maps (F) for CBF, CBV, and MTT- ROI (white arrow) placed in the ischemic penumbra, with mirror ROI (black arrow) in the contralateral hemisphere. rCBF and rCBV values are higher than the pre-thrombolysis values (left hand bottom corner of each map). However, there is no significant change in the size of the penumbra as compared to the prethrombolysis perfusion CT

The role of NCCT, CTA, and CTP is highlighted in the illustrative example in Figure 8.

A simple algorithm proposed by Tomandal[10] for quick assessment is given in Table 4.

**Table 4:** Assessment of CT perfusion parameters

Pathologic condition	TTP	CBF	CBV
None	Normal	Normal	Normal
Arterial stenosis with excellent compensation	Prolonged	Normal	Normal
Oligemic tissue	Prolonged	(>60%)	(>80%)
Tissue at risk	Prolonged	(>30%)	(>60%)
Tissue irreversibly damaged	Not measurable	(<30%)	(40%)

## CTP *vs.* MRI Perfusion

MRI perfusion and diffusion imaging provide similar information with greater sensitivity and specificity.[14] However, there are certain inherent advantages of CT scan–based imaging. CT scan is more widely available as compared to MRI and thus imaging can be done earlier. It is relatively less expensive. Also, evaluation of the carotids, intracerebral vessels, and cerebral perfusion can be done in a very short time: approximately 5 min. The major disadvantage of CT scan is the exposure to ionizing radiation and the inability of present-day scanners to image the entire brain for perfusion imaging.[11]

The American Heart Association has provided certain guidelines and recommendations for imaging of cerebral ischemia. According to it, quantitative CTP may probably be useful to differentiate between reversible and irreversible ischemic tissue in acute stroke patients (strength of recommendation: grade c). On the other hand, MRI perfusion and diffusion techniques are probably useful in differentiating between reversible and irreversible ischemic tissue in acute stroke patients (strength of recommendation: grade b).[15]

Thus, both techniques may be used, although at present MRI perfusion has more evidence in its favor. More blinded studies with randomization are required before more specific recommendations can be made. Still, in our setting, CTP remains a valuable and important tool in stroke imaging.
